# Negative regulation of root-knot nematode parasitic behavior by root-derived volatiles of wild relatives of *Cucumis metuliferus* CM3

**DOI:** 10.1093/hr/uhac051

**Published:** 2022-02-28

**Authors:** Xiaoxiao Xie, Jian Ling, Zhenchuan Mao, Yan Li, Jianlong Zhao, Yuhong Yang, Yanlin Li, Mingyue Liu, Xingfang Gu, Bingyan Xie

**Affiliations:** 1 College of Horticulture, Hunan Agricultural University, Changsha 410128, China; 2 Institute of Vegetables and Flowers, Chinese Academy of Agricultural Science, Beijing 100081, China

## Abstract

Root-knot nematodes (RKN; *Meloidogyne* spp.) cause a significant decrease in the yield of cucumber crops every year. *Cucumis metuliferus* is an important wild germplasm that has resistance to RKN in which plant root volatiles are thought to play a role. However, the underlying molecular mechanism is unclear. To investigate it, we used the resistant *C. metuliferus* line CM3 and the susceptible cucumber line Xintaimici (XTMC). CM3 roots repelled *Meloidogyne incognita* second-stage larvae (J2s), while the roots of XTMC plants attracted the larvae. CM3 and XTMC were found to contain similar amounts of root volatiles, but many volatiles, including nine hydrocarbons, three alcohols, two aldehydes, two ketones, one ester, and one phenol, were only detected in CM3 roots. It was found that one of these, (methoxymethyl)-benzene, could repel *M. incognita*, while creosol and (*Z*)-2-penten-1-ol could attract *M. incognita*. Interestingly, creosol and (*Z*)-2-penten-1-ol effectively killed *M. incognita* at high concentrations. Furthermore, we found that a mixture of CM3 root volatiles increased cucumber resistance to *M. incognita*. The results provide insights into the interaction between the host and plant-parasitic nematodes in the soil, with some compounds possibly acting as nematode biofumigation, which can be used to manage nematodes.

## Introduction

Root-knot nematodes (RKN, *Meloidogyne* spp.) are considered the most important plant-parasitic nematodes in various economic crops [1, 2]. These parasites are obligatory endoparasites inhabiting the roots, where they cause structural, physiological, and biochemical changes in the host plants [3]. Among the well-known plant-parasitic nematodes, *Meloidogyne incognita*, *M. javanica*, *M. arenaria*, and *M. hapla* are the most common and damaging species [3]. *M. incognita* is highly invasive and has spread to 143 countries [4].

Under natural conditions, the host factors involved in attracting or repelling nematodes are extremely complicated. Root volatiles of different crops or different varieties can influence the host preference of RKN in different ways [5, 6]. So far, more than 10 root compounds have been reported to affect *M. incognita* movement in vegetables [5–9]. For example, methyl salicylate, 2-isopropyl-3-methoxypyrazine and tridecane are attractive to second-stage larvae (J2) of nematodes [5, 7]. On the other hand, thymol derived from pepper root, either alone or combined with other pepper root volatiles, repels root-knot, cyst, and stubby root nematodes [7]. When *M. incognita* was exposed to phenol, 4-methylphenol, γ-decalactone, and skatole, they all demonstrated nematicidal activity with low median lethal concentration (LC50) values [9]. Knowledge of these root compounds can be used to develop alternative and sustainable methods for management of RKN. However, the relationship between root volatiles and nematodes in cucurbits has not yet been reported.

Cucumber (*Cucumis sativus* L., 2*n* = 2*x* = 14) is a popular commercial vegetable crop grown around the world [10]. Cucumber is an ideal host to RKN species, including *M. incognita*, which can cause severe plant damage and yield losses [11]. Unfortunately, there are currently few options available for RKN control. There are three wild species that have been confirmed to be *M. incognita*-resistant: *Cucumis metuliferus* Naud., *C. hystrix* Chakr., and *C. melo* var. *texanus* [12–16]. It has been shown that *M. incognita* J2s are less able to penetrate the roots of *C. metuliferus*, and they induce smaller giant cells and produce fewer eggs, compared with cucumber [14, 16–18]. Although it is an important RKN-resistant resource for cucurbitaceous crops, little is known about the mechanism underlying this resistance. Understanding the mechanisms underlying *C. metuliferus* resistance to RKN may help us to develop efficient control of pest nematodes in Cucurbitaceae crops. Therefore, studying why *C. metuliferus* repels nematodes and whether there are any substances that can help cucumber avoid nematode infection or kill nematodes around roots is of great significance to cucumber production. In this study, we first assessed the chemotaxis of *M. incognita* J2s to the root tips of the cucumber line Xintaimici (XTMC) and the *C. metuliferus* line CM3. In addition, we also tested the chemotaxis of *M. incognita* to total root volatiles released by XTMC and CM3. We then identified three volatile substances from CM3 roots and characterized their chemotaxic and nematicidal activities. Finally, we tested whether CM3 root volatiles can protect cucumber roots from *M. incognita* infestation.

## Materials and methods

### Plant materials and nematode population

Cucumber inbred line Xintaimici (XTMC) and *C. metuliferus* inbred line CM3 were provided by the Department of Cucurbits Genetics and Breeding, Institute of Vegetable and Flowers (IVF), Chinese Academy of Agricultural Science (CAAS), Beijing, China. XTMC and CM3 were grown in a glass room of a phytotron in September 2020 with day/night temperatures 28/18°C, 85–100% relative humidity, and a day length of ~14 hours. One-month-old plants were used for the experiments.

Egg masses of *M. incognita* were extracted from infected roots of water spinach (*Ipomoea aquatica* Forssk.) in Beijing, China (116.3°E, 39.9°N) and placed in plastic culture plates (90 mm diameter × 15 mm height) with distilled water to hatch at 28°C for 2–7 days. J2s that emerged from eggs were counted using a stereomicroscope before use in the bioassays.

### Inoculation assays

To evaluate *M. incognita* infection of XTMC and CM3, galls and egg masses were counted for three biological replicates consisting of 10 plants each. The plants were grown in plastic pots filled with vermiculite and peat. Each plant was inoculated with 1000 J2s. Approximately 45 days after inoculation, each plant was uprooted and rinsed free of soil. Roots were placed in plates with water to count galls and egg masses. The fresh weight of the root of each plant was measured.

### Behavioral response of *M. incognita* J2 nematodes to root tips

Nematodes can move on the surface of water agar [7, 8, 19]. Water agar (0.5%) was prepared and poured into the wells of a six-well cell culture plate. After cooling, ~1-cm root tips were spread on the surface of the water agar. The horizontal distance between the root tips of the two materials was 1 cm. Two hundred J2 nematodes were spotted at a distance of 1 cm from each of the root tips of the two materials. The placement of root tips and nematodes is shown in Fig. 2a. The plates were placed in the dark for 6 hours at 28°C and were then photographed under a microscope and the number of nematodes gathered around the root tips was counted.

### Assays of chemotaxis of J2 nematodes to root odors

Roots of CM3 and XTMC seedlings were carefully removed from the plastic pots and gently washed three times with sterile water. They were then transferred to a sterilized mortar and ground into a puree for later use. Water agar (0.5%) was poured into one of three sections of compartmentalized Petri dishes. Two hundred J2s were concentrated into 5 μl and spotted on the water agar surface. Root puree (0.35 g) was placed in each of the other two compartments. The distance from the nematode to the root tissues on both sides was 2 cm. The placement of nematodes and root tissue in the Petri dish is shown in Fig. 3a. The Petri dish was then covered with a lid. After 10 hours in the dark, the number of nematodes found around the root tissue was observed and recorded. The area where the number of nematodes was counted is shown in the shaded part of Fig. 3a. The width of the shaded area was 1 cm. The response of *M. incognita* to CM3 and XTMC root odors was evaluated as described in Fig. 3a.

### Volatile analysis

Volatile analysis was carried out as previously described with slight modifications [20, 21]. A DVB/CAR/PDMS solid-phase microextraction (SPME) fiber (Supelco Inc., PA, USA) and an Agilent 7890B-5977A GC–MS with a split–splitless injector were used in this test. The SPME fiber needs to be conditioned at 250°C for 30 min before use. The roots were gently pulled out of the substrate and washed carefully with tap water. Then the roots were rinsed with sterile water. We absorbed the water on the root surface with sterilized filter paper. The cleaned root system was frozen in liquid nitrogen and ground into powder. Two grams of pulverized root tissue and 0.6 g of NaCl were mixed in a 20-ml glass vial with a screw cap (Agilent, CA, USA). We added 2050 ng of 2-nonanone to the glass vial as an internal standard. Subsequently, the glass vial was put into a 50°C water bath for 10 minutes with agitation.

To collect root volatiles, An SPME fiber was cleaned in the GC injection port for 10 minutes at 250°C. Then, the SPME fiber was moved into the headspace of the root ground sample for a 40 min extraction. After extraction, the SPME fiber was quickly inserted into the injector of the Agilent 7890B-5977A GC–MS to desorb the extract for 15 minutes at 250°C. The GC–MS program followed the method of Wang *et al*. method [22].

Volatile compounds were identified by comparing the collected mass spectra with the spectra in the National Institute for Standards and Technology (NIST 14) data bank. The relative content of volatile components was determined with the area normalization method. Volatile compounds of roots were determined as nanograms per gram of fresh weight following the equation: volatile compound (A) content in root (ng/g, fresh weight) = peak area (A) × 1025(2-nonanone, ng/g)/peak area (2-nonanone), where A stands for any identified compound in roots, and 2-nonanone is the synthetic standard. The experiment was repeated three times.

**Figure 1:**
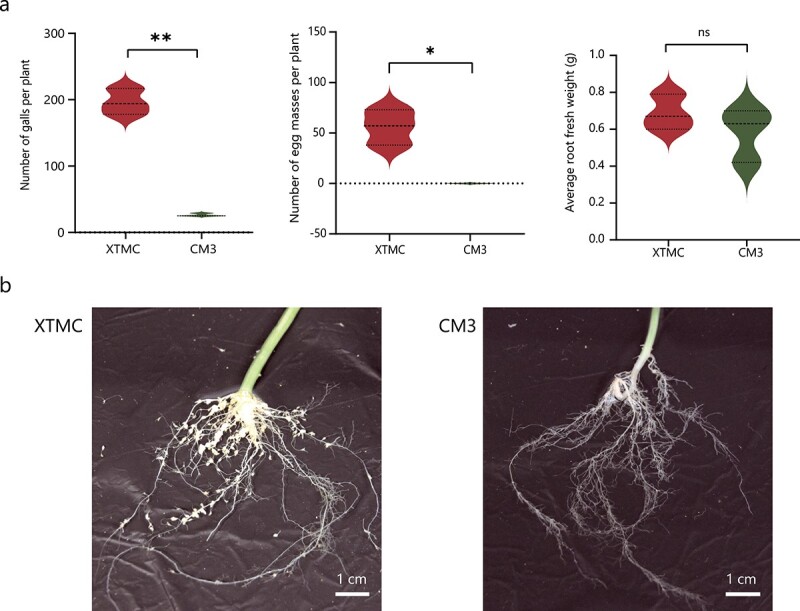
Symptoms and responses of *M. incognita* J2 nematodes on susceptible XTMC and resistant CM3. **a** Number of galls, number of egg masses, and root fresh weight per plant of XTMC and CM3 at 45 days post-inoculation. Data are presented as means ± standard deviation. ^*^P < .05; ^**^P < .01; ns, not significant (Student’s *t*-test). **b** Roots of XTMC and CM3 at 45 days post-inoculation.

### Bioactivity of identified compounds

Among the 18 volatiles unique to CM3 roots, (methoxymethyl)-benzene (CAS: 538–86-3), creosol (CAS: 93-51-6), and (*Z*)-2-penten-1-ol (CAS: 1576-95-0) were diluted into a 4000 ng/μL stock solution in distilled water containing 2% (v/v) methanol. The stock solution was serially diluted three times to make 1000, 250, and 62.5 ng/μl concentrations and then used in assays for chemotaxis and nematicidal activity. For chemotaxis analysis, a swab of cotton was placed in each of two cells of the three-cell Petri dish and 0.5% water agar was poured into the third cell. About 200 J2s in 5 μl were placed in the middle of the water agar. One milliliter of compound and 1 ml of 2% methanol in distilled water (control) were spotted on the cotton in the two grids. After 6 hours in the dark, the number of nematodes near each of the cotton swabs was counted under a stereo microscope. Similarly, to identify nematicidal activity, a total of 2 ml of compound was added to the cotton on both sides. After sealing the Petri dish for 48 hours, clean water was sprayed on the water agar surface and nematodes were allowed to recover in the water for 10 minutes. The numbers of living and dead nematodes were counted to estimate nematicidal activity. Each trial comprised four replicates, and each replicate contained three plates.

Methyl salicylate (≥99%), (methoxymethyl)-benzene (≥99%), creosol (≥98%), and (*Z*)-2-penten-1-ol (≥98%) were purchased from Shanghai Macklin Biochemical Technology Co., Ltd.

**Figure 2:**
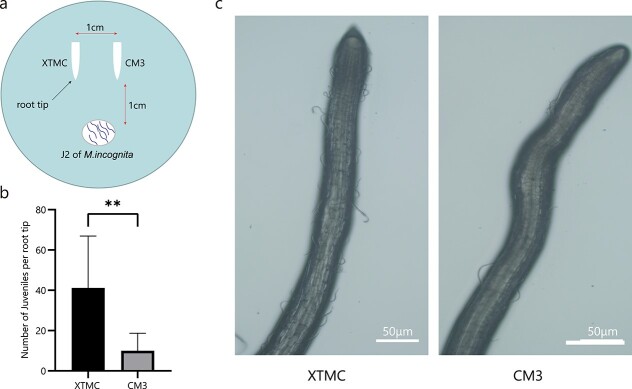
Behavioral response of J2 nematodes to XTMC and CM3 root tips. **a** Schematic representation of XTMC and CM3 root tips for *in vitro* chemotaxis assays. **b** Comparison of the number J2s per root tip of XTMC and CM3 at 6 hours post-inoculation. Data are presented as means ± standard deviation (*n* = 33). ^**^P < .01 (Wilcoxon signed rank test). **c** J2s around root tips of XTMC and CM3 at 6 hours post-inoculation.

### Effects of CM3 root volatiles on *M. incognita* infection of cucumber

A double-pot culture device (shown in Fig. 5a) was used to study the effect of CM3 root volatiles on *M. incognita* infection of cucumber. The device had upper and lower nutrient bowls. The bottom of the upper nutrient bowl was removed and three layers of 200-mesh filter screens were laid to allow gas to pass through and prevent roots from passing through. In the test group, three CM3 plants were planted in the lower nutrient bowl and in the control groups there were three XTMC plants or no plants in the lower nutrient bowl. The upper nutrient bowl was filled with the substrate. The entire device was sealed to balance the gas in the internal space of the device. Three days later, one cucumber (XTMC) seedling was planted in the upper nutrient bowl of the test group and the control group. After 3 days, 700 nematodes were inoculated in the upper nutrient bowl. Twenty days after inoculation, the number of root galls of each cucumber seedling planted in the upper nutrient bowl was counted. The test consisted of three replicates with 4–10 plants in each replicate.

### Statistical analysis

All data were recorded using Microsoft Excel 2017. Statistical analyses were performed using GraphPad Prism and R software.

## Results

### Comparison of root symptoms of CM3 and XTMC in response to *M. incognita* infection

Numbers of galls and egg masses and plant fresh weight were used as measures of susceptibility/resistance of plants to *M. incognita* (Fig. 1a). By 45 days after inoculation with *M. incognita* the average number of root galls formed on CM3 roots was ~26 and no egg masses were visible. In contrast, the average numbers of galls and egg masses found on XTMC roots were 196 and 56, respectively. The average fresh weight of XTMC (0.69 g) and CM3 (0.58 g) did not differ significantly (*t* = 1.025, df = 4, *P* = .3631). The number of galls on CM3 was significantly lower than that on XTMC (*t* = 14.92, df = 4, *P* < .0001), and the number of egg masses showed the same trend (*t* = 5.536, df = 4, *P* = .0052). Our results clearly demonstrate that CM3 is highly resistant to *M. incognita* compared with the susceptible control, XTMC (Fig. 1b).

### Movement and selection of *M. incognita* is host genotype-dependent

To assess nematode preference, root tips of CM3 and XTMC were placed apart on the surface of 0.5% water agar in a cell culture dish, as shown in Fig. 2a. J2 nematodes were then placed on the agar surface equidistant from the two roots, and the plates were incubated in the dark. After 6 hours, the number of J2 worms gathered around the root tips was counted. About four times as many (*P* < 0.01) nematodes gathered around the root tips of XTMC (~41) compared with CM3 (~10) root tips (Fig. 2b and c). *M. incognita* nematodes thus exerted a strong preference for XTMC roots.

### Chemotactic effects of CM3 and XTMC root volatiles on *M. incognita*

**Figure 3:**
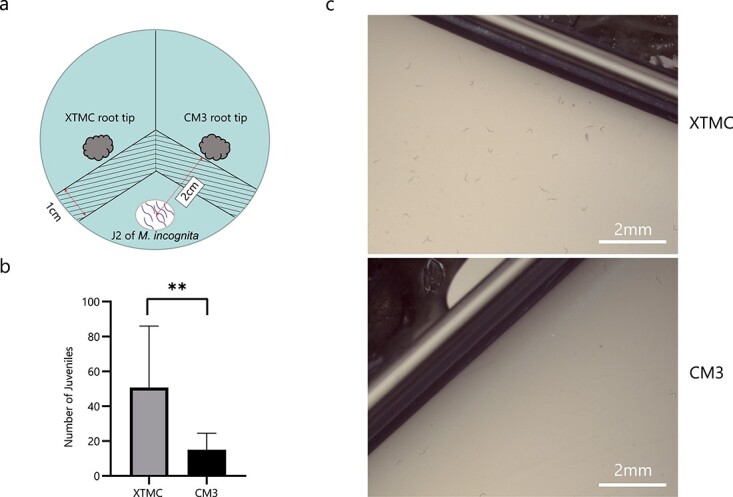
Assays of chemotaxis J2 nematodes to root odors. **a** Schematic representation of root odors for *in vitro* chemotaxis assays. **b** Comparison of the number of J2s attracted to root odors of XTMC and CM3 at 8 hours post-inoculation. Data are presented as means ± standard deviation (*n* = 33). ^**^P < .01 (Wilcoxon signed rank test). **c** J2s attracted via root odors of XTMC and CM3 at 8 hours post-inoculation.

To further test whether volatiles from CM3 and XTMC roots affect the behavior of J2 nematodes, the chemotaxis of *M. incognita* nematodes in response to the two root volatiles was tested in a three-compartment Petri dish. The experimental device used is shown in Fig. 3a. Plates containing J2 nematodes placed on 0.5% water agar were incubated in the dark for 10 hours, and the position of the nematodes relative to the roots was used as an indication of their preference. Fewer nematodes were found near the roots of CM3 than near XTMC roots: ~15 versus ~50, respectively (Fig. 3b and c; *P* < .001), which further supports the strong preference of *M. incognita* for XTMC roots*.*

### Volatile compounds in roots of CM3 and XTMC

**Figure 4:**
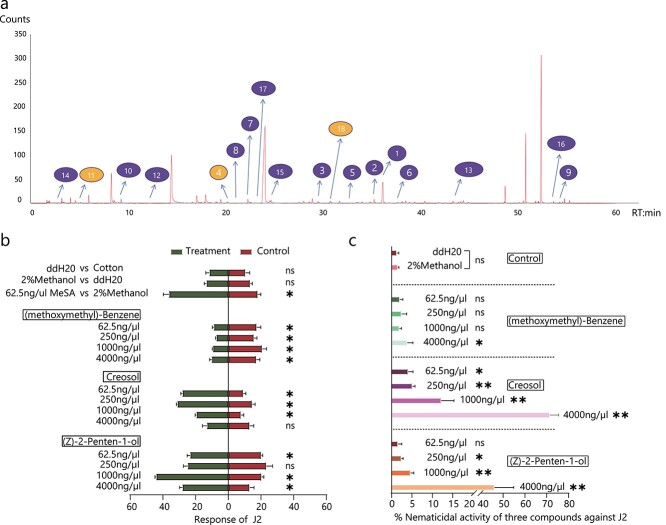
GC–MS chromatogram analyses identified CM3-derived volatile compounds that show different chemotaxic and nematicidal activity for *M. incognita*. **a** Gas chromatogram obtained from SPME sampling of the CM3 root system. Peaks labeled 1–18 represent compounds that were identified only in CM3 roots, and not in XTMC roots. (Methoxymethyl)-benzene (4), creosol (18), and (Z)-2-penten-1-ol (11), marked in yellow, were used in follow-up tests; **b** Chemotaxis assays of J2s with different concentrations of (methoxymethyl)-benzene, creosol, and (*Z*)-2-penten-1-ol compared to controls. ^*^P < .05; ns, not significant (Wilcoxon signed rank test). Data are presented as means ± standard deviation. **c** Nematicidal activity of different concentrations of (methoxymethyl)-benzene, creosol, and (*Z*)-2-penten-1-ol. ^*^P < .05; ^**^P < .01; ns, not significant (Welch’s *t*-test). Data are presented as means ± standard deviation.

GC–MS analysis of the plant headspace volatiles identified a total of 101 compounds, including 86 compounds in CM3 roots and 83 compounds in XTMC roots (Supplementary Data Table S1). Of these, 68 compounds were found in roots of both genotypes, while the following18 compounds were only found in CM3 roots: nine hydrocarbons, three alcohols, three aldehydes, two ketones, an ester, and a phenol (Fig. 4a; Table 1). Overall, the most abundant volatile compound was 3-(1-methylethenyl)-cyclooctene (CAS: 61233-78-1), which was present at 113.25 ng/g. The next most abundant compounds were 2,4-dimethyl-1-(1-methylethenyl)-cyclohexene (CAS: 56763-60-1), 2-isopropylidene-3-methylhexa-3,5-dienal (CAS: 1000191-76-5), 4-tetradecyne (CAS: 60212-33-1), and creosol (CAS: 93-51-6), which were present at 17.93, 13.24, 11.82, and 11.61 ng/g, respectively. Among the 18 compounds, eight had concentrations >5 ng/g. A total of 15 compounds (10 alcohols, 2 aldehydes, 2 hydrocarbons, and 1 ester) were found to be exclusive to XTMC roots (Supplementary Data Table S2). Six of these compounds had concentrations >5 ng/g, including methyl salicylate (CAS: 119-36-8), which has been reported as a chemoattractant for *M. incognita* [7, 19].

**Table 1:** Detailed information on volatile compounds unique to the root system of CM3

Rank	Volatile compound (number)	Content (ng/g FW^a^)	CAS^b^	RT^c^/min
	**Hydrocarbons (9)**			
1	3-(1-Methylethenyl)-cyclooctene	113.25 ± 5.74	61 233-78-1	36.11
2	2,4-Dimethyl-1-(1-methylethenyl)-cyclohexene	17.93 ± 1.01	56 763-60-1	35.22
3	4-Tetradecyne	11.82 ± 1.10	60212-33-1	29.50
**4**	**(Methoxymethyl)-benzene**	**9.37** ± **1.04**	**538-86-3**	**20.16**
5	2-Ethenyl-1,1-dimethyl-3-methylene-cyclohexane	2.65 ± 0.15	95 452-08-7	32.69
6	1-Chloro-5-methyl-hexane	1.95 ± 0.21	33 240-56-1	36.77
7	1-Nonyne	1.19 ± 0.02	3452-09-3	22.65
8	*Trans*-β-ocimene	0.26 ± 0.03	3779-61-1	20.67
9	1-Octadecyne	0.11 ± 0.00	629-89-0	54.33
	**Alcohols (3)**			
10	1-Hexanol	8.50 ± 0.35	111-27-3	9.30
**11**	**(*Z*)-2-penten-1-ol**	**1.75** ± **0.12**	**1576-95-0**	**5.14**
12	1,1,1-Trichloro-2-propanol	1.01 ± 0.08	76-00-6	12.30
	**Aldehydes (2)**			
13	2-Isopropylidene-3-methylhexa-3,5-dienal	13.24 ± 0.82	1 000 191-76-5	43.30
14	3-Methyl-butanal	1.24 ± 0.02	590-86-3	2.94
	**Ketones (2)**			
15	3a,4,5,7a-Tetrahydro-3a,6-dimethyl-,*cis*-(.+/−.)-2(3H)-benzofuranone	7.91 ± 0.19	33 722-72-4	28.58
16	6,10,14-Trimethyl-2-pentadecanone	0.49 ± 0.20	502-69-2	53.93
	**Esters (1)**			
17	11-Dodecyn-1-ol acetate	1.16 ± 0.19	53 596-78-4	23.27
	**Phenols (1)**			
**18**	**Creosol**	**11.61** ± **1.41**	**93-51-6**	**30.74**

Compounds in bold type indicate use in chemotaxis assay.

a Root fresh weight.

b Chemical Abstract Service number.

c Retention time.

### Differential responses of *M. incognita* to specific compounds

To determine *M. incognita* responses to specific volatiles from CM3 roots, we conducted preference tests for 3 of the 18 unique compounds. Creosol and (*Z*)-2-penten-1-ol have been reported to repel insects [23–25]. Hydrocarbons accounted for the largest proportion. So we selected one compound, (methoxymethyl)-benzene, as the third compound for further analysis. The results are shown in Fig. 4b. The J2 nematodes showed obvious rejection of (methoxymethyl)-benzene at all concentrations tested (*P* < .05), but showed a preference for creosol at concentrations of 62.5–1000 ng/μl (*P* < .05). At concentrations of 1000 ng/μl and 4000 ng/μl (*Z*)-2-penten-1-ol was preferred by J2 nematodes compared with the control (*P* < .05). In summary, all concentrations of (methoxymethyl)-benzene tested showed strong repulsion of the nematodes, while creosol and (*Z*)-2-penten-1-ol attracted nematodes at certain concentrations.

### Nematicidal activity of synthetic versions of volatile compounds present in CM3 roots

To test the nematicidal activity of the three compounds, nematodes were placed in Petri dishes containing four different concentrations of the compounds and incubated for 48 h (Fig. 4c). The results showed that the rates of nematode survival in the distilled water (ddH_2_O) and 2% methanol treatments were not significantly different. (Methoxymethyl)-benzene did not affect nematode survival at concentrations between 62.5 and 1000 ng/μl compared with 2% methanol, while concentrations of 4000 ng/μl had a small effect on mortality rate (3.85%). Creosol promoted nematode mortality at all concentrations tested, with higher concentrations having stronger effects, killing 12.18% of nematodes at 1000 ng/μl creosol and 71.43% at 4000 ng/μl. Similarly, concentration-dependent effects on nematode mortality were seen for of (*Z*)-2-penten-1-ol, with the strongest effect seen at 4000 ng/μl.

### Effects of natural CM3 volatiles on cucumber infestation by *M. incognita*

**Figure 5:**
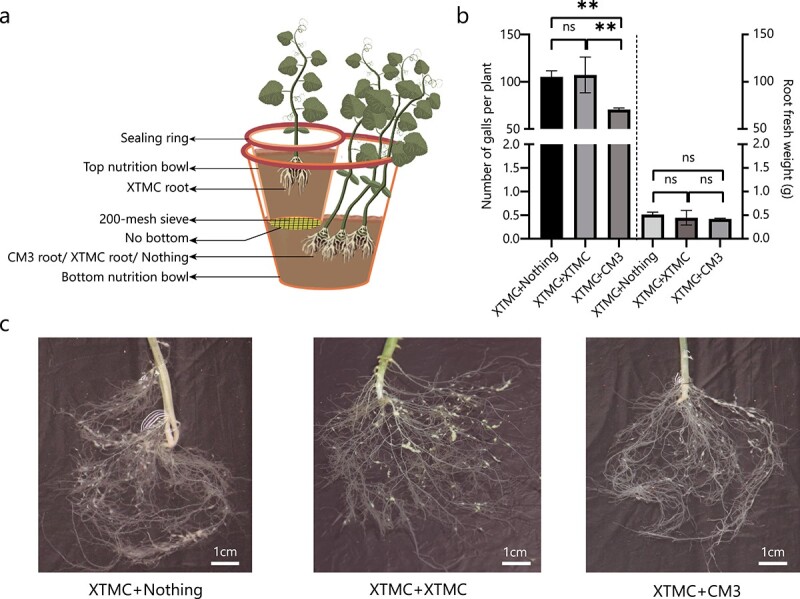
Effects of natural CM3 root volatiles on cucumber infestation by *M. incognita*. **a** Schematic representation of the double-pot culture experiment. **b** Gall number and root fresh weight per plant of XTMC (top bowl) + nothing (bottom bowl), XTMC (top bowl) + XTMC (bottom bowl), and XTMC (top bowl) + CM3 (bottom bowl) at 20 days post-inoculation. Data are presented as means ± standard deviation. ^**^P < .01; ns, not significant (one-way ANOVA following Dunnett’s *post hoc* test). **c** Roots of XTMC seedlings growing in the three devices at 20 days post-inoculation.

To detect the effect of volatiles from CM3 roots on cucumber infection by *M. incognita*, we used a sealed, double-layered culture device as shown in Fig. 5a. Twenty days after inoculation, the cucumber seedlings in the upper nutrient bowl were investigated. No differences were observed between the root fresh weights of the experimental group and the two control cucumber seedlings. The average number of galls on cucumbers in the test group was ~70, compared with 105 [XTMC (top bowl) + nothing (bottom bowl); *P* = .0024] and 107 [XTMC (top bowl) + XTMC (bottom bowl); *P* = .0016] in the two control groups.

## Discussion

### *C. metuliferus* resistance to *M. incognita*: preventing nematode infection and nematode development

The numbers of galls and egg masses per root system can be used to assess nematode infection and reproduction on a plant [3, 26]. In this study, we systematically compared the resistance of CM3 and XTMC to *M. incognita*. Our findings for RKN infection are consistent with previous studies [27–30]. Compared with cucumber, *C. metuliferus* showed high resistance to RKN, which is manifested in a smaller number of galls and inability to generate egg masses. Under the experimental conditions used, only a handful of J2 nematodes were able to successfully infect the root system of CM3, and the few nematodes that were able to infect the plant were unable to generate visible egg masses by 45 days after inoculation. *C. metuliferus* achieves resistance to RKN using two strategies: (i) preventing nematodes from infecting roots; and (ii) reducing the fecundity of the nematodes that do infect the roots.

### Root volatiles may be important factors underlying the resistance observed in *C. metuliferus*

The recognition and response of nematodes to host roots is a complex process involving multiple stages. But nematodes can be attracted to roots and germinating seeds by smelling or tasting a series of compounds [31, 32], allowing them to locate and infect a suitable host in the soil. Previous studies indicate that the movement of nematodes in the soil is affected by volatile and soluble components in the rhizosphere [5, 7, 8, 33].

The results of previous experiments showed that *C. metuliferus* is an important resource for resistance to RKN [17, 30, 34, 35]. However, there are very few studies on its resistance mechanism. In this study, we found a strong preference in J2 nematodes for cucumber (XTMC) over *C. metuliferus* (CM3) root tips. Studies using ground root tips verified that *M. incognita* prefers XMTC to CM3. In the latter assay partitions between the tissues and nematodes ensure that the only signal that is emitted and received is gas. The nematodes are thus responding to root odors, suggesting that root volatile compounds help the non-feeding J2 nematodes to direct their movements. Moreover, we examined *C. metuliferus* root volatiles and studied the effects of three specific volatiles on *M. incognita* infection. In addition, the results of the double-pot culture experiment indicated that the root volatiles of CM3 had the potential to help cucumber root to resist *M. incognita* infection. Because of the limitations of the equipment used, the results need to be verified later in more tests. For example, barrier materials between the top nutrient bowls and bottom nutrient bowls that allow gas to pass through and prevent nematodes from passing through can be used to replace 200-mesh sieves in the device. In conclusion, root volatiles may be one of the important factors enabling *C. metuliferus* to develop resistance to *M. incognita*.

### Potential *M. incognita* biofumigation in *C. metuliferus* roots

The compounds emitted from host plant roots play a vital role in the process of nematode infestation of the host. In recent decades, there have been many studies on volatiles in host roots and their repellency to nematodes, with studies carried out on more than a dozen host plants [5, 6, 8, 36–43]. These chemical compounds have potential to be used in biofumigation to control pest nematodes. For instance, plant volatiles emitted from *Brassica* species have been reported to control plant-parasitic nematodes by incorporating green plant debris into the soil [44–46]. Important volatile compounds – isothiocyanates (ITCs) – from many *Brassica* family plants are known to control *M. javanica* in field experiments [47]. Indeed, ITCs as active ingredients can be found among synthetic commercial nematicidal formulations [48].

This is the first study on volatile compounds in the roots of cucurbitaceous crops. Two cucurbitaceous materials were selected for this test: one was the RKN-resistant African horned melon (*C. metuliferus*, CM3), and the other was the susceptible *C. sativus* (‘Chinese Long’, XTMC). Using GC–MS analysis we identified 18 unique volatiles in CM3 roots. In previous studies, many compounds have been confirmed to have attractive or repellent effects on nematodes, but these 18 compounds are not among them. This may be due to the different crops being tested. The most abundant unique volatile substance detected was 3-(1-methylethenyl)-cyclooctene. However, the relationship between this compound and nematode infestation requires further study. Among these volatiles, creosol (2-methoxy-4-methylphenol) is recognized as an important insect repellent and can control trypanosomosis in cattle [23, 24]. Another of the identified compounds, (*Z*)-2-penten-1-ol, has biological activity on spider mites and is a potential insect repellent [25]. By classification, hydrocarbons accounted for the largest proportion of the unique compounds. Among the nine hydrocarbons identified we chose (methoxymethyl)-benzene, creosol, and (*Z*)-2-penten-1-ol, for use in subsequent studies of chemotaxis and nematicidal activity.

We found that (methoxymethyl)-benzene was repellent to *M. incognita* and may be one of the reasons that nematodes are unwilling to parasitize CM3. Although creosol is also unique to CM3 roots, it attracted nematodes at lower doses, and its nematicidal activity only becomes strong at higher doses. This interesting result shows that some substances emitted from the roots of CM3 may have a deceptive effect on the nematodes. We hypothesize that the nematodes are attracted to the host at low concentrations, and, as they approach the host, the nematodes are gradually exposed to high concentrations of compounds and die. This interesting hypothesis provides a new idea to consider for the prevention and control of nematodes in production: attracting nematodes in order to kill them. Of course, this can be implemented as a combination of one or more substances. The third test compound chosen for study was an alcohol compound, (*Z*)-2-penten-1-ol. The effect of this chemical on nematodes was similar to that of creosol. In summary, many volatiles are produced by *Cucumis* roots, which, depending on their concentrations, can exert chemotaxic activities. This suggests that the effect of host root volatiles on the parasitic behavior of nematodes is complex. In addition, the performance of these root volatiles has potential for use in nematode control.

## Acknowledgements

This work was supported by the National Natural Science Foundation of China (No. 31571996), the Key R&D Projects of Inner Mongolia (2020GG0110), the National Key R&D Projects (2017YFD020060, 2018YFD0201204), Hainan Yazhou Bay Seed Laboratory (B21HJ0214), the Natural Science Foundation of Hunan Province (2019JJ50280), the Science and Technology Innovation Program of the Chinese Academy of Agricultural Sciences (CAAS-ASTIP-2017-IVF), and the Earmarked Fund for Modern Agro-industry Technology Research System (CARS-23).

## Author contributions

X.X. designed this study, performed the experiments, analyzed the data, and wrote the manuscript. J.L. analyzed the data and contributed to revisions of the manuscript. B.X. and X.G. designed the study and revised the manuscript. M.Z.,L.Y. and Y.Y. performed some experiments of the manuscript. Z.J.,L.Y. and L.M. revised the manuscript. All authors read and approved the final manuscript.

## Data availability

All relevant data can be found within the paper and its supporting materials.

## Conflict of interest

The authors declare no competing interests.

## Supplementary data

Supplementary data is available at *Horticulture Research* online.

## Supplementary Material

Web_Material_uhac051
