# Supplementary material for: Negative regulation of root-knot nematode parasitic behavior by root-derived volatiles of wild relatives of Cucumis metuliferus CM3
Source: Hortic Res. 2022 Feb 28;9:uhac051. doi: 10.1093/hr/uhac051 (PMC9071375; doi:10.1093/hr/uhac051)
Supplement: Web_Material_uhac051 [file web_material_uhac051.zip › table s2.doc]

TableS2. Detailed information on volatile compounds unique to the root system of XTMC

| Rank | Volatile compounds (number) | content (ng/gFW^a^) | CAS^b^ | RT/min |
| --- | --- | --- | --- | --- |
|  | **Alcohols (10)** |  |  |  |
| 1 | Benzyl alcohol | 21.03±6.92 | 100-51-6 | 19.83 |
| 2 | Bicyclo[3.1.1]hept-2-ene-2-methanol, 6,6-dimethyl- | 19.29±1.83 | 515-00-4 | 30.86 |
| 3 | Bicyclo[3.1.1]heptan-3-ol, 6,6-dimethyl-2-methylene-, [1S-(1.alpha.,3.alpha.,5.alpha.)]- | 11.81±0.60 | 547-61-5 | 26.68 |
| 4 | 4,8-Decadien-3-ol, 5,9-dimethyl- | 3.15±0.24 | 67845-54-9 | 32.72 |
| 5 | 2-Octyn-1-ol | 2.47±0.37 | 20739-58-6 | 21.32 |
| 6 | Bicyclo[2.2.1]heptane-2,5-diol, 1,7,7-trimethyl-, (2-endo,5-exo)- | 2.46±0.08 | 10359-41-8 | 28.73 |
| 7 | Bicyclo[2.1.1]hexan-2-ol, 2-ethenyl- | 2.25±0.56 | 1000221-37-2 | 10.24 |
| 8 | endo-Borneol | 2.02±0.20 | 507-70-0 | 28.64 |
| 9 | Cyclohexanol, 2,2-dimethyl- | 1.98±0.21 | 1193-46-0 | 29.57 |
| 10 | 11-Tridecyn-1-ol | 1.78±0.11 | 33925-75-6 | 23.26 |
|  | **Hydrocarbons (2)** |  |  |  |
| 11 | Tridecane | 7.76±2.12 | 629-50-5 | 38.01 |
| 12 | Benzene, 1-ethenyl-4-methoxy- | 3.01±0.33 | 637-69-4 | 27.85 |
|  | **Aldehydes (2)** |  |  |  |
| 13 | 2,6-Octadienal, 3,7-dimethyl-, (E)- | 29.42±0.51 | 141-27-5 | 36.02 |
| 14 | 1-Cyclohexene-1-carboxaldehyde, 4-(1-methylethenyl)- | 5.41±0.59 | 2111-75-3 | 31.37 |
|  | **Esters (1)** |  |  |  |
| **15** | **Methyl salicylate** | **15.86±1.94** | **119-36-8** | **30.67** |

FW^a^: Root Fresh Weight.

CAS^b^: Chemical Abstract Service Number.

Bold compound was used for chemotaxis.
